# Normative Data of the EORTC QLQ-C30 For the German Population: A Population-Based Survey

**DOI:** 10.1371/journal.pone.0074149

**Published:** 2013-09-10

**Authors:** Annika Waldmann, Daniel Schubert, Alexander Katalinic

**Affiliations:** 1 Institute of Social Medicine and Epidemiology, University of Luebeck, Luebeck, Germany; 2 Institute of Cancer Epidemiology, University of Luebeck, Luebeck, Germany; Queensland University of Technology, Australia

## Abstract

**Aim:**

The aim of the present study was to generate up-to-date normative data for health-related quality of life (QoL) measured with the “European Organization for Research and Treatment of Cancer (EORTC) Core Quality of Life Questionnaire (QLQ-C30)” in a random sample of the population in Northern Germany.

**Methods:**

We conducted a population-based survey of a random sample of 10,000 persons aged 16 years or older. The postal questionnaire included questions regarding lifetime prevalence of common diseases and quality of life. EORTC QLQ-C30 scales were scored according to standard procedures. The results were stratified for age and sex.

**Results:**

The questionnaire was completed by 4,684 (47%) of 9,928 eligible persons. Mean age of the participants was 51.7 years (standard deviation: 18.5) and 57% were females. Missing values for the EORTC QLQ-C30 scales and items were sparse (minimum: 0.2%, maximum: 1.5%). Self-reported health related QoL varied by age and sex. Generally, men reported better functioning and fewer symptoms than women. In both sexes function declined and symptoms increased with increasing age. Symptoms most frequently reported were fatigue, pain and insomnia. Compared to the German reference data published in 2001 our participants scored more than 10 points higher on the latter three scales/items. The most frequently reported diseases were hypertension (36%), hyperlipidemia (26%) and arthritis (30%). Lifetime prevalence of depression was 16% in women and 11% in men.

**Conclusion:**

Our study participants are representative for the German general population with regard to age, sex and education. Of special interest is the high proportion of participants reporting depression which is also mirrored by high fatigue, pain and insomnia scores. The normative data provided should be used as comparison health-related QoL data when evaluating the QoL in German cancer patients.

## Introduction

Recent estimates for Germany indicate that the five-year prevalence of cancer patients is approximately 1.33 million [1]. Improved treatment and early detection regimes have contributed to an increased survival of cancer patients – at least in the well-developed countries. As survival has improved for most cancer types, the impact of disease and treatment on patient’s quality of life (QoL) becomes more important in clinical practice as well as in health care research and clinical research [2]. During the last decades several questionnaires to assess QoL have been developed. In oncology one of the most frequently used questionnaires to measure health-related quality of life is the Quality of Life Questionnaire Core module with 30 items provided by the European Organization for Research and Treatment of Cancer (EORTC QLQ-C30). The QLQ-C30 is a multi-dimensional assessment tool as it includes a range of items covering physical, emotional und social health issues – issues being relevant to cancer patients irrespective of diagnosis. It can be used for detection of early and long-term consequences of the cancer disease itself and its treatment. Further, the QLQ-C30 can be used for individual patient management as well as for evaluation of undesired treatment effects on the group level. There is sufficient evidence to support its reliability and validity [3,4].

Although asking patients about their QoL is clinically intuitive, simple and easy to conduct, a number of methodological problems are to be discussed. In particular, the question which values in a continuum of possible values between 0 and 100 indicate ‘a good quality of life’ is very challenging [2,5]. Osoba et al. raised the question of clinical importance of differences and defined differences of 10 points or more as clinically relevant [6] – both for differences within and between persons and groups, respectively. Another problem is that “baseline values” in cancer patients are collected after the persons have been diagnosed with cancer. At this time cancer patients may be already psychologically affected by the cancer diagnosis and may suffer from symptoms – both biases baseline values. Therefore, using reference data from the general population of corresponding age and sex is an important alternative to assess the baseline QoL of individual patients [5,7].

The EORTC QLQ-C30 has been translated into more than 60 languages. It is said to be applicable across a range of cultural settings [8]. However, Hjermstadt emphasizes the importance of national reference data [9] as quality of life measured with the EORTC QLQ-C30 was found to vary by age and sex. Furthermore, QoL seems to be influenced by the cultural context and/or the degree of morbidity in the general population [5,10,11]. Therefore, national reference data can help researchers and clinicians to interpret their patients’ self-assessed QoL.

The most recent reference / normative data for the EORTC QLQ-C30 was published for samples of the Swedish [7] and the Dutch population [12]. In 2001, Schwarz and Hinz published reference data for Germany based on a sample of 2,028 persons [13]. However, these data were collected by means of face-to-face interviews in Leipzig, Eastern Germany, nine years after the reunification. At that time marked social and health differences were found between Western and Eastern Germany, e.g. the proportion of unemployed persons in Eastern Germany was nearly twice as high as in Western Germany and the proportion of persons at-risk-for-poverty was 7% higher in Eastern than in Western Germany. Further, life expectancy differed by 2 years for women and 3 years for men and morbidity patterns showed relevant differences. Today social differences between the “old” and the “new” federal states are still to be found – even though to a lesser extent [14,15]. Thus, it can be questioned whether the Schwarz and Hinz data still reflect the situation in Germany today. As new data are warranted, we aimed to assess up-to-date normative data for the EORTC QLQ-C30 in a random sample of the population in Luebeck, Northern Germany, aged 16 years and older with our survey “33 questions for Luebeck”.

## Materials and Methods

### Ethics Statement

Regardless of age, each participant gave written consent to study participation on behalf of his/her own person. The study protocol included a description of and justification for this approach. The ethics committee of the University of Luebeck approved the study protocol (reference number 11-245).

### Population-based survey

The population-based survey “33 questions for Luebeck” consisted of two survey periods: 5,000 persons were contacted in spring (March–April) and in summer (August–September), respectively. The residents’ registration office of the city of Luebeck (213,000 inhabitants), Northern Germany, provided names, addresses, birthdates and sex for a random sample of 10,000 persons with a minimum age of 16 years (as at February 2012). The sample was representative with regard to age, sex and urban districts.

Persons were sent a letter informing about the study, the questionnaire and a stamped return envelope. If a person did not provide an answer within four weeks, the study material was sent once again to the potential study participant. The questionnaire included the validated German version of the EORTC QLQ-C30 (version 3.0), questions on employment and education status as well as on acute or chronic diseases (lifetime prevalence; question “Has a physician ever told you that you have one of the following diseases?” followed by a list of diseases).

### EORTC QLQ-C30

The EORTC QLQ-C30 (version 3.0) was designed and validated to assess health-related QoL considering the previous week [3]. It is a patient self-rating questionnaire that can be applied to all cancer patients. The EORTC QLQ-C30 is composed of a global health status/QoL-score, five (multi-item) function scales (physical, role, social, emotional and cognitive functions), three multi-item symptom scales (fatigue, pain, nausea), and five single items (dyspnea, insomnia, appetite loss, constipation, diarrhea). A final item evaluates the perceived economic consequences of the disease. Each item has four response alternatives: (1) “not at all”, (2) “a little”, (3) “quite a bit”, and (4) “very much” – except the two items of the global health-status/quality of life scale which have response options ranging from (1) “very poor” to (7) “excellent”.

According to the guidelines provided by the EORTC all scores of the QLQ-C30 were transformed linearly so that all scales ranged from 0 to 100. In the function scales higher scores represent a better level of functioning while in the case of symptom scales/items higher scores mark a higher level of symptomatology or problems. Missing responses were handled according to the manual, i.e. if at least half of the items from a particular scale have been answered by a study participant, the missing item was replaced with the average of the items of the corresponding scale [4]. Differences between subgroups were interpreted in accordance with the suggestion of Osoba et al. who defined differences of 10 points or more as clinically relevant [6].

### Statistical analyses

Given the huge number of study participants which would lead to significant test results even in the presence of only small differences, statistical testing for difference in mean values or distribution was not conducted.

Qualitative data was described with absolute and relative frequencies, quantitative data with means and standard deviations. Respondents were compared to non-respondents and active deniers with regard to age, sex and urban district (defined by zip codes) in a descriptive manner. QoL data are presented for the respective age by sex groups. Data on co-morbid diseases are given as absolute and relative frequencies.

## Results

### Study participants

**Figure 1:**
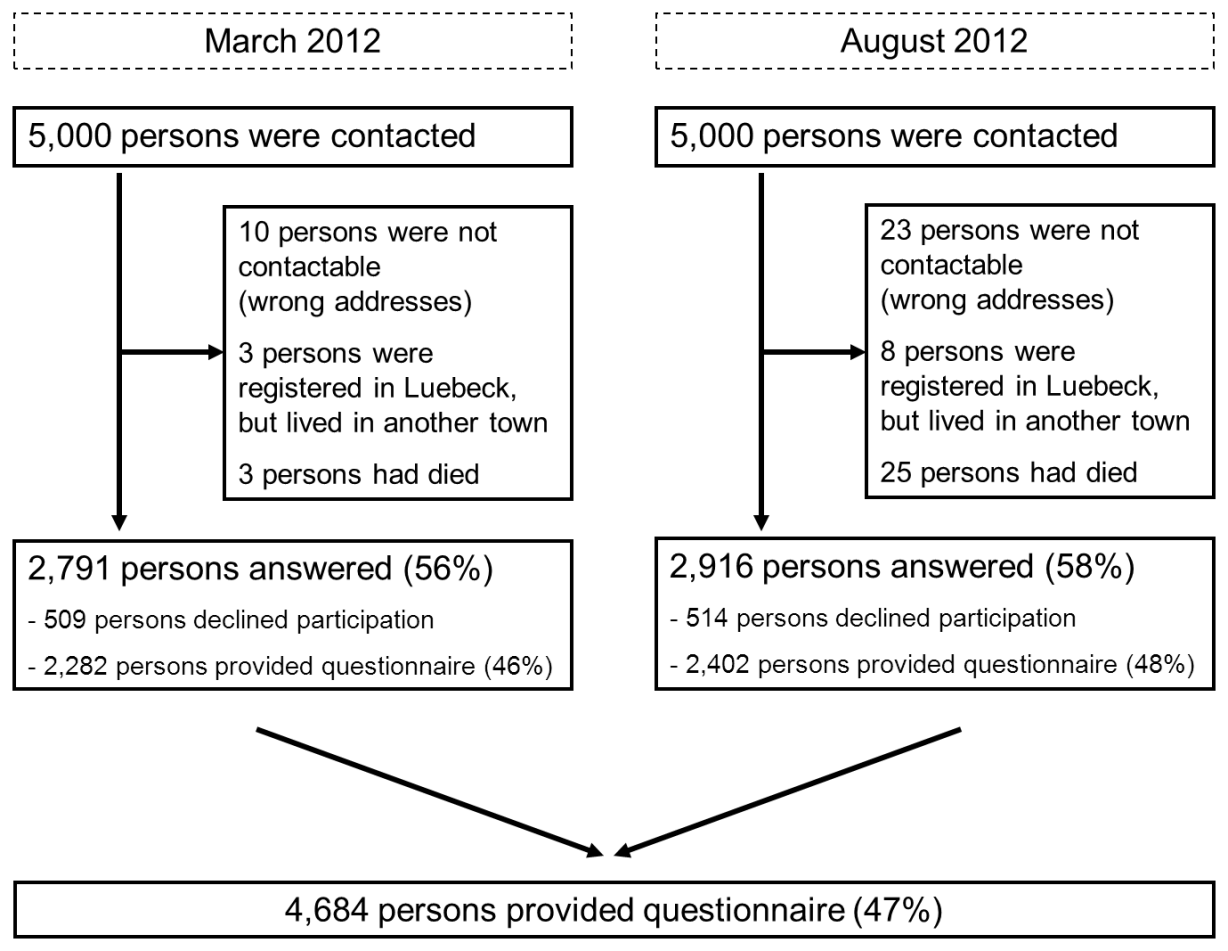
Flow Chart.

The residents’ registration office provided data for a representative sample of 10,000 persons living in the city of Luebeck. The sample had a mean age of 48.4 years (SD: 18.7) and 51.6% of the sample were women. After excluding 72 persons who had died, had an unknown address or were still registered in Luebeck but not living there (16/5,000 in March 2012; 56/5,000 in August 2012), 9,928 persons (51.6% women) were eligible for the study (Figure **1**).

The overall response proportion was 57.5%. Women (62.6%) were more likely to respond to the study invitation than men (52.1%). But as 1,023 respondents actively declined study participation, the participation proportion was somewhat lower (47.2%, n=4,684). Overall, 2,634 (51.4%) women and 2,050 men (42.7%) sent back the self-administered questionnaire. For each woman that declined participation 4.6 women participated in the study and 3.4 women did not respond. In contrast, for each man that declined study participation 4.5 men participated and 5.1 men did not respond.

**Figure 2:**
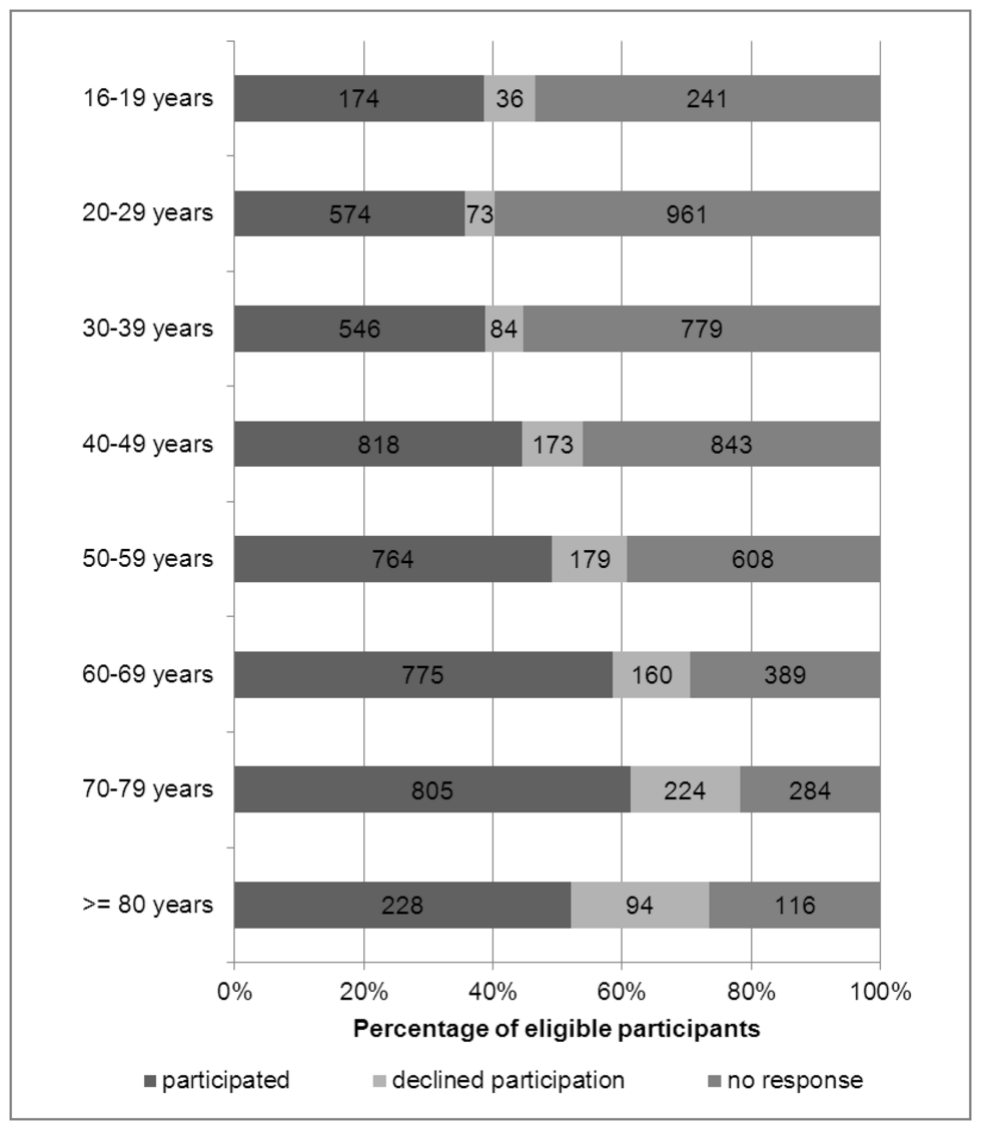
Participation according to age groups (numbers in bars are absolute numbers).

Not only sex but also age influenced study participation. Until the age up to 39 years, about 1/3 of the contacted persons participated in our study while more than 50% of those aged 60 or older did so. Active decline of study participation was most frequently seen in the oldest age groups with sometimes family members or carers stating that the contacted persons would not participate due to health reasons. Mean age of all study participants was 51.7 years (SD: 18.5), thereby three years higher than in the total sample of 10,000 persons (Figure **2**). Urban district had no influence on participation behavior as active deniers, non-responders and participants showed a similar distribution with regard to urban districts.

Characteristics of study participants stratified by sex can be found in Table **1**. Data on self-reported diseases (lifetime prevalence) are reported in Table **2** as crude frequencies. Hypertension, arthritis and hyperlipidemia were the three co-morbidities most often reported by women and men. About 16% of women and 11% of men reported a diagnosis of depression of which each 43% also reported another mental disease such as anxiety or psychosis. Lifetime prevalence of any malignancy was approximately 8% in women and men.

**Table 1:** Baseline description of study participants (absolute and (relative frequencies)).

	Women [n=2,634]	Men [n=2,050]	Women - general population	Men - general population
Mean age + SD	51.4 + 18.5	52.2 + 18.5	-	-
Age groups1				
16-19 years	81 (3.1)	93 (4.5)	5.5%	6.1%
20-29 years	348 (13.2)	226 (11.0)	13.4%	14.7%
30-39 years	315 (12.0)	231 (11.3)	13.3%	14.3%
40-49 years	489 (18.6)	329 (16.0)	18.3%	20.1%
50-59 years	415 (15.8)	349 (17.0)	16.3%	17.1%
60-69 years	413 (15.7)	362 (17.7)	12.7%	12.6%
70-79 years	438 (16.6)	367 (17.9)	12.5%	10.8%
>= 80 years	135 (5.1)	93 (4.5)	8.0%	4.2%
Education2				
No educational level/degree	32 (1.2)	24 (1.2)	4.1%	3.9%
9 years (compulsory school;“Volksschule/Hauptschule”)	817 (31.5)	624 (31.0)	40.0%	39.7%
10 years (“Realschule/Polytechnische Oberschule”)	851 (32.8)	557 (27.7)	24.4%	19.9%
12-13 years (university entrance degree)	850 (32.8)	765 (38.0)	30.4%	35.7%
Other	42 (1.3)	42 (2.1)	0.9%	0.7%
Occupation				
Studying (school, university)	150 (6.2)	140 (7.2)	-	-
Employed (worker, employee)	1607 (66.5)	1149 (58.7)	-	-
Self-employed	167 (6.9)	203 (10.4)	-	-
Civil servant / public officer	116 (4.8)	220 (11.2)	-	-
Other	376 (15.6)	244 (12.5)	-	-

1 Data Source for general population: Bevölkerungsanteile und gesamte Bevölkerung im Jahresdurchschnitt 2011. Fortschreibung des Bevölkerungsstandes, Statistisches Bundesamt (URL: http://www.gbe-bund.de/gbe10/i?i=19D, last accessed 5th July 2013).

2 Data Source for general population: Bevölkerung mit Angaben zum allgemeinen Schulabschluss in 1000 von 2005 bis 2009. Mikrozensus 2009 (URL: http://www.gbe-bund.de/gbe10/i?i=636D, last accessed 5^th^ July 2013)

**Table 2:** Self-reported diseases in the study population according to sex (lifetime prevalence; absolute and relative frequency).

	Women [n=2,634]		Men [n=2,050]	
	N (%)		N (%)	
Hypertension	885 (33.6)		785 (38.3)	
Hyperlipidemia/Hypercholesterolemia	632 (24.0)		559 (27.3)	
Cardiovascular disease	222 (8.4)		330 (16.1)	
Cancer	219 (8.3)		157 (7.7)	
Diabetes mellitus	214 (8.1)		222 (10.8)	
Respiratory disease (e.g. bronchial asthma, allergic asthma, COPD, chronic bronchitis, pulmonary emphysema)	372 (14.1)		281 (13.7)	
Gastritis, ulcus	327 (12.4)		232 (11.3)	
Renal disease (renal stones, impaired renal function, nephritis, pyelonephritis)	228 (8.7)		171 (8.3)	
Arthritis/Arthrosis (e.g. knee, hip)	823 (31.2)		519 (25.3)	
Inflammatory arthropathy (e.g. rheumatoid arthritis, *Morbus Bechterew*)	332 (12.6)		206 (10.0)	
Osteoporosis	240 (9.1)		64 (3.1)	
Depression	435 (16.5)		240 (11.7)	
Other mental diseases (e.g. anxiety, psychosis)	254 (9.6)		144 (7.0)	

### QoL

Overall, the proportion of missing values for the EORTC QLQ-C30 scales and items was low ranging from 0.2% to 1.5% in women and from 0.6% to 1.3% in men. In the total group of participants men scored slightly higher on the function scales and women reported a slightly higher level of symptomatology/problems (Tables **3** and **4**). In general, younger persons scored higher on the function scales and lower on the symptom scales/items than did the elderly (Tables **3** and **4**; see also supplementary e-files).

**Table 3:** Normative data for women according to age.

	All women [n=2,634]		16-19 yrs [n=81]	20-29 yrs [n=348]	30-39 yrs [n=314]	40-49 yrs [n=488]	50-59 yrs [n=414]	60-69 yrs [n=412]	70-79 yrs [n=436]	>=80 yrs [n=135]
	N	Mean (SD)	Mean	Mean	Mean	Mean	Mean	Mean	Mean	Mean
Global quality of life scale	2,595	65.4 (23.9)	71.0	71.1	69.2	67.9	63.2	65.5	60.9	49.5
Physical function	2,623	83.6 (20.5)	92.0	92.8	91.6	89.8	82.6	81.0	73.1	58.3
Role function	2,616	78.1 (29.8)	91.5	85.6	84.0	82.7	75.0	77.3	71.0	54.9
Emotional function	2,620	67.2 (26.5)	66.4	64.2	65.7	64.7	62.2	72.8	72.7	67.6
Cognitive function	2,620	83.7 (22.6)	85.6	85.6	85.6	83.1	81.3	87.1	81.9	78.1
Social function	2,616	81.3 (28.0)	90.9	88.5	82.5	81.5	78.4	83.1	78.7	66.0
Fatigue	2,623	33.7 (26.2)	35.2	33.4	31.8	31.3	34.1	29.2	37.0	49.2
Nausea and vomiting	2,622	4.8 (13.1)	5.3	6.0	4.5	4.1	5.7	3.7	4.3	6.3
Pain	2,629	30.2 (31.4)	19.5	21.1	23.5	25.8	33.1	31.6	38.6	50.7
Dyspnea	2,617	16.8 (26.7)	8.8	9.2	8.0	12.1	18.8	18.9	25.8	36.6
Insomnia	2,622	31.4 (33.8)	22.2	22.2	23.9	27.0	38.5	34.1	37.7	43.8
Appetite loss	2,618	9.1 (20.7)	9.1	11.7	7.2	7.5	9.4	7.0	9.1	18.0
Constipation	2,621	8.5 (21.3)	7.0	5.2	6.2	4.9	9.1	7.9	13.6	19.3
Diarrhea	2,617	9.6 (21.3)	11.5	10.3	9.6	8.4	9.2	9.0	10.3	10.9
Financial difficulties	2,608	13.0 (26.7)	5.4	6.1	10.3	12.8	17.1	14.0	14.7	21.3

**Table 4:** Normative data for men according to age.

	All Men	[n=2,050]	16-19 yrs [n=93]	20-29 yrs [n=226]	30-39 yrs [n=230]	40-49 yrs [n=329]	50-59 yrs [n=350]	60-69 yrs [n=360]	70-79 yrs [n=367]	>=80 yrs [n=93]
	N	Mean (SD)	Mean	Mean	Mean	Mean	Mean	Mean	Mean	Mean
Global quality of life scale	2,023	67.4 (23.4)	79.9	74.6	71.7	65.6	65.2	65.9	65.0	55.6
Physical function	2,033	87.0 (19.0)	95.2	94.8	95.3	91.1	88.9	83.0	79.5	63.1
Role function	2,030	80.7 (27.9)	90.0	90.8	87.7	81.7	80.4	78.6	74.9	57.8
Emotional function	2,027	72.4 (24.5)	80.3	70.7	70.5	67.5	69.8	75.7	76.6	71.8
Cognitive function	2,029	83.8 (21.8)	90.1	86.9	86.9	84.6	83.6	85.1	78.9	73.8
Social function	2,027	83.3 (26.4)	93.5	91.0	87.6	83.7	82.9	80.6	79.7	68.9
Fatigue	2,033	28.8 (24.9)	23.5	25.8	24.9	28.6	29.6	27.3	31.7	43.2
Nausea and vomiting	2,033	3.5 (11.4)	4.7	3.4	3.6	4.3	3.9	2.2	3.0	4.2
Pain	2,038	25.2 (29.1)	14.9	16.5	17.9	24.6	29.3	26.5	29.5	39.9
Dyspnea	2,032	15.3 (26.0)	5.4	4.7	7.3	11.2	14.0	20.9	24.3	33.3
Insomnia	2,030	23.7 (31.0)	12.2	20.4	16.6	24.4	28.6	25.9	25.3	24.3
Appetite loss	2,032	7.6 (19.3)	11.5	6.2	5.4	8.0	8.3	7.3	6.8	12.7
Constipation	2,031	6.0 (18.0)	3.2	2.2	2.6	5.4	4.9	5.5	9.7	19.6
Diarrhea	2,023	9.5 (21.1)	7.2	10.1	8.0	13.4	9.7	8.0	8.8	7.3
Financial difficulties	2,023	12.0 (25.8)	3.6	5.0	8.0	10.8	14.0	15.3	15.2	18.3

### Differences between survey phases

Response and participation rate were slightly higher in late summer than in spring 2012 (Figure **1**). The proportion of women among the participants was comparable between the two survey periods (spring: 55.9% women; summer: 56.6% women), but mean age of participants differed by four years (spring: 50 years, summer: 54 years). Accordingly, age-related diseases such as arthritis (27 vs. 31%) and cancer (7 vs. 9%) as well as hypertension (34 vs. 38%) and hyperlipidemia/hypercholesterolemia (24 vs. 28%) were more frequently reported in summer. Nevertheless, differences between QoL scales in the total groups of respondents at the two time points were small with a range from -2 to +2.

## Discussion

Our survey aimed to assess up-to-date normative data for the EORTC QLQ-C30 in a random sample of a population in Luebeck, Northern Germany, with a minimum age of 16 years. Four important findings emerge from our study: First of all, although we contacted a random sample of the “apparently healthy” population, study participation was remarkably high with 47% of eligible persons filling out the questionnaire. Participation behavior followed the expected pattern with women compared to men being more likely to participate and younger as well as the oldest persons compared to middle-aged persons being less likely to respond. Second, a high level of self-reported morbidity was found in our random sample of the population. Especially the prevalence of a “lifetime diagnosis” of mental diseases was high. Third of all, QoL differences between men and women and younger and older persons, respectively, were as expected – that is, men scored slightly higher on the function scales, women reported a slightly higher level of symptomatology/problems and younger persons scored higher on the function scales and lower on the symptom scales/items than the elderly. And finally, if differences of ten points or more were considered as clinically relevant as suggested by Osoba and colleagues [6], relevantly higher levels of fatigue, pain and insomnia were found when our data was compared to (age-adjusted) Schwarz and Hinz data published in 2001 [13].

Cross-sectional studies (surveys) are easy to realize at comparably low costs. But several problems are inherent in this study type: One problem of cross-sectional studies is their “snapshot” character, that is, surveys are often conducted only at one time point or period. Another problem is associated with the mode of recruitment and the resulting representativeness of the respondents. With regard to representativeness random samples are to be preferred in contrast to -for example- volunteers answering to advertisements. However, random samples hold the danger of low response rates as there is no relationship (of whatever nature) between potential participants and researchers resulting – depending on the number of potential participants – in small subgroups. In our survey we contacted 10,000 potential participants – that is 5.5% of the total population living in Luebeck aged 16 years or older [16]. The sample was representative with regard to age, sex and urban district. The latter had no influence on study participation while younger persons and men were less likely to respond. This participation pattern was also found in the study of Derogar et al. [7] and of van de Poll-Franse et al. [12]. Nevertheless, study participation in our study was very high allowing subgroup analyses. The high participation rate might be due to the context. In the year 2012, Luebeck was the City of Science (“Stadt der Wissenschaft” [17]) – a title that is awarded by the Donors’ Association for German Science (“Stifterverband für die Deutsche Wissenschaft” [18]). In the context of the “City of Science” several events, awareness campaigns and public campaigns were conducted with the aim of bringing science, researchers and the public together. This and the wish to contribute to a good reputation of the city may have prompted a high motivation to participate.

Current data regarding the health status or morbidity of the Luebeck population is not available in detail, therefore, we compare our data to the German DEGS-sample (Studie zur Gesundheit Erwachsener in Deutschland (*DEGS*) [German Health Interview and Examination Survey for Adults]: data collection: 11/2008 -01/2012; 8,152 participants, Germany-wide recruitment, 180 study centres [19]) and to the GEDA-2010-sample (Gesundheit in Deutschland aktuell (GEDA) [German Health Update]: GEDA complements DEGS; data collection: 09/2009 -07/2010, telephone interview with 22,050 participants, Germany-wide recruitment [20]).

Regarding the diseases of affluence our data corresponds well to the DEGS- and GEDA-2010 data. Hypertension, arthritis and hyperlipidemia were the three diseases most often reported in our study. In the GEDA-sample the single most prevalent health condition was hypertension (32% in women, 31% in men; lifetime prevalence) followed by hyperlipidemia (women: 28%; men: 31%) and arthritis (women: 27%, men: 18% [20]). A diabetes diagnosis was reported by 9.5% of our participants which is a little higher than the percentage of GEDA-participants (women: 9%, men: 8% [20]) and DEGS-participants with known diabetes (7.2%; taking diabetes medication or reporting a medical diagnosis of diabetes). If self-reported levels of blood glucose and/or HbA1c were taken into account an additional 0.7-2.1% of DEGS-participants (depending whether either glucose or HbA1c or both were considered) were classified as having diabetes [19].

Approximately 8% of women and men in our sample had survived a tumor disease which equals the lifetime prevalence for any malignant disease in the female GEDA-2009-sample (8.4%) while GEDA-men reported a lifetime prevalence of 5.3% [21]. On the one hand, the difference in lifetime prevalence of any cancer might be due to the slightly differing age structure between our and the GEDA-2009-sample (proportion of study participants aged >= 50 years: 53.9% of men in our sample, 43.1% in the GEDA-sample), with our sample being older. On the other hand, it might be due to the fact that the federal state of Schleswig-Holstein is one of the states in Germany with highest incidences for prostate cancer, malignant melanoma, basal and squamous cell carcinoma [22]. Not only prostate cancer, but also non-melanoma skin cancer has a high five-year relative survival rate which might have contributed to the higher lifetime prevalence of any malignant tumor disease in our cohort.

Regarding the mental disorders a higher degree of morbidity was found in our sample, as 14.7% of participants state to have a lifetime diagnosis of depression while only 8.1% of the DEGS-sample show symptoms of depression within the past 14 days (assessed with PHQ-9 [19]) and only 9.0% of women and 5.1% of men had a diagnosis of depression within the last 12 months in the GEDA-sample [20] (data of the GEDA-2009-sample: 8.0% of women and 4.5% of men [21]). Differences in study design (lifetime prevalence vs. prevalence with past 12 months and past 14 days, respectively) and general time trends are partly responsible for the observed differences. Not only for Germany, but also for Europe it was recently shown that the number of persons with mental disorders has been increasing during the last 25 years [22].

In conclusion, our study sample –drawn from a random sample of the population- shows a morbidity pattern that seems to reflect the frequency and the morbidity pattern in the current German population with the exception of a slightly increased prevalence of mental diseases. This level of morbidity has to be kept in mind when QoL data of “healthy” reference population are compared to QoL data of well-defined groups of patients.

The differences in EORTC QLQ-C30 scores (between men and women, young and old persons) found in our study as well as in other studies [7,12,13] underline the ability of the EORTC QLQ-C30 to detect differences in health-related QoL not only in the original target group –that is in cancer patients- but also in the general population. Nevertheless, a random sample of the general population will include healthy persons as well as diseased persons. As can be assumed, clinically relevant differences in QoL were found in our sample for ill and healthy persons (*data not shown in detail*).

QoL data of our German study participants stratified by sex and age groups is different from recently published QoL data of a Dutch [12] and a Swedish [7] general population sample which might be due to either cultural reasons or linguistic differences between the various translations of the EORTC QLQ-C30 as discussed by Scott and colleagues [10]. In comparison to data from The Netherlands [12] and Sweden [7] global QoL/health status in our study is clinically relevantly lower for the age groups 50-59, 60-69, and 70 years and older and also somewhat lower in the younger age groups of our study population.

Compared to the Schwarz and Hinz data published in 2001 [13] the global QoL/health status reported by our study participants is lower for all age groups except for women aged 60 to 69 years. When the age structure of our sample is used to age-adjust the Schwarz and Hinz data [13] differences for the global QoL/health status (difference of 0.2 points in women and of 1.3 points in men) and for most of the other scales and items tended to be small and not clinically relevant – with the exception of the domains fatigue (Schwarz and Hinz women: 18.9, men: 11.0), insomnia (women: 18.7, men: 13.3) and pain (women: 18.6, men: 13.5). The high level of mental diseases in our cohort corresponds well to the higher levels of fatigue, insomnia and pain in our study participants – symptoms for which co-occurrence has been shown and which were also shown to be associated with mental disorders [16,23,24]. Although the aforementioned differences between the older and the new reference data are small, we suggest to use the latest normative data as they reflect the current situation in Germany better than the data gathered in the end of the 1990s [15]. For instance, the proportion of unemployed persons in Germany today is about 6.6% with 10.3% in Eastern Germany, 5.8% in Western Germany and 6.8% in Schleswig-Holstein [25]. The proportion of persons having less than 60% of the median German equivalent income (“at-risk-of-poverty”) differed by 7% in 1990 and by 6.4% today (Germany today: 14.4%, Eastern Germany: 19.5%, Western Germany: 13.1%, Schleswig-Holstein: 13.1%) [26]. Life expectancy and morbidity pattern approximated for Eastern and Western Germany. However, data for Schleswig-Holstein (mean values, proportions) are in most cases very much alike the data for total Germany [14,15]. Therefore, we recommend the utilization of our data as normative data e.g. in the context of health care research, clinical studies or certification processes of cancer centers. Files for age adjustment of our normative data can be found in the supporting information section (see supplementary e-files; Table S1 and Table S2).

Our survey has some strengths and limitations. As discussed earlier we experienced a quite high response rate – given the recruiting method and addressing a randomly drawn sample. A total number of 174 study participants aged 16 to 19 years and 228 participants aged 80 years or older are sufficient to provide reliable estimates for groups often underrepresented in study samples. However, all our study participants were recruited in a 213,000 citizen city located in Northern Germany. Although the participants’ age and sex distribution is comparable to that in total Germany, regional differences regarding QoL scoring within Germany cannot be ruled out with certainty.

## Conclusions

Our study participants are representative for the German general population with regard to age, sex and education. Of special interest is the high proportion of participants reporting depression which is also mirrored by high fatigue, pain and insomnia scores. The up-to-date normative data provided should be used as comparison health-related QoL data when evaluating the QoL in German cancer patients.

## Supporting Information

Table S1 **Interactive Excel Sheet for age adjustment of the normative data (5-years age categories).**
(XLS)

Table S2 **Interactive Excel Sheet for age adjustment of the normative data (10-years age categories).**
(XLS)
